# The impact of a disease management programme for type 2 diabetes on health-related quality of life: multilevel analysis of a cluster-randomised controlled trial

**DOI:** 10.1186/s13098-018-0330-9

**Published:** 2018-04-10

**Authors:** Sigrid Panisch, Tim Johansson, Maria Flamm, Henrike Winkler, Raimund Weitgasser, Andreas C. Sönnichsen

**Affiliations:** 1 Department of Mathematics, University of Salzburg, Hellbrunner Str. 34, 5020 Salzburg, Austria; 2 Institute of General Practice, Family Medicine and Preventive Medicine, Paracelsus Medical University, Salzburg, Strubergasse 21, 5020 Salzburg, Austria; 3 Paris Lodron University, Kapitelgasse 4-6, 5020 Salzburg, Austria; 4 Department of Internal Medicine, Wehrle-Diakonissen Hospital, Haydnstrasse 18, 5020 Salzburg, Austria; 5 Paracelsus Medical University, Strubergasse 21, 5020 Salzburg, Austria; 6 Institute of General Practice and Family Medicine, Witten/Herdecke University, Alfred-Herrhausen-Straße 50, 58448 Witten, Germany; 7 Division of Population Health, Health Services Research and Primary Care, School of Health Sciences, University of Manchester, Oxford Rd, Manchester, M13 9PL UK

**Keywords:** Cluster randomised controlled trial, Type 2 diabetes mellitus, Disease management programme, Health related quality of life (EQ-5D-3L questionnaire), Multilevel modeling, Sex-specific analysis

## Abstract

**Background:**

Type 2 diabetes is a chronic disease associated with poorer health outcomes and decreased health related quality of life (HRQoL). The aim of this analysis was to explore the impact of a disease management programme (DMP) in type 2 diabetes on HRQoL. A multilevel model was used to explain the variation in EQ-VAS.

**Methods:**

A cluster-randomized controlled trial—analysis of the secondary endpoint HRQoL. Our study population were general practitioners and patients in the province of Salzburg. The DMP “Therapie-Aktiv” was implemented in the intervention group, and controls received usual care. Outcome measure was a change in EQ-VAS after 12 months. For comparison of rates, we used Fisher’s Exact test; for continuous variables the independent T test or Welch test were used. In the multilevel modeling, we examined various models, continuously adding variables to explain the variation in the dependent variable, starting with an empty model, including only the random intercept. We analysed random effects parameters in order to disentangle variation of the final EQ-VAS.

**Results:**

The EQ-VAS significantly increased within the intervention group (mean difference 2.19, p = 0.005). There was no significant difference in EQ-VAS between groups (mean difference 1.00, p = 0.339). In the intervention group the improvement was more distinct in women (2.46, p = 0.036) compared to men (1.92, p = 0.063). In multilevel modeling, sex, age, family and work circumstances, any macrovascular diabetic complication, duration of diabetes, baseline body mass index and baseline EQ-VAS significantly influence final EQ-VAS, while DMP does not. The final model explains 28.9% (EQ-VAS) of the total variance. Most of the unexplained variance was found on patient-level (95%) and less on GP-level (5%).

**Conclusion:**

DMP “Therapie-Aktiv” has no significant impact on final EQ-VAS. The impact of DMPs in type 2 diabetes on HRQoL is still unclear and future programmes should focus on patient specific needs and predictors in order to improve HRQoL.

*Trial registration* Current Controlled trials Ltd., ISRCTN27414162

**Electronic supplementary material:**

The online version of this article (10.1186/s13098-018-0330-9) contains supplementary material, which is available to authorized users.

## Background

The prevalence of type 2 diabetes is increasing and the social and economic burden of this disease grows rapidly [1, 2]. Guideline adherent, structured treatment and management of the disease as proposed by DMPs are the best strategies to prevent acute and chronic complications, while preserving a good quality of life [3, 4]. Several studies show that the quality of life in diabetes is decreased compared to individuals without diabetes [5] and HRQoL decreases with disease progression and complications [6, 7]. Predictive factors for HRQoL are age, sex, low socioeconomic status (family and work circumstances, level of education, residential area), ethnicity, obesity, comorbidities, and any macrovascular diabetic complication [5, 7–12]. The prevalence of type 2 diabetes is higher among women than among men. A recent meta-analysis demonstrated that women are at higher risk of developing both coronary heart disease and stroke compared to men [13, 14]. Increasing knowledge concerning HRQoL in diabetic patients, as well as the predictors, is crucial in improving diabetes management. Overall, evidence from RCTs and systematic reviews on DMPs reveal only modest effects on patient care [3, 4], especially regarding improvement in clinically relevant endpoints such as HRQoL. We previously demonstrated that the Austrian DMP “Therapie-Aktiv” implemented by statutory health insurance improves process quality and enhances weight reduction, but did not significantly improve metabolic control (HbA1c) [15]. Participants in the German DMP for type 2 diabetes rated their HRQoL in the dimensions mobility, self-care and performing usual activities higher compared to patients in routine care [16]. Although studies are reporting the effect of DMPs on quality of life in diabetes patients [17–20], none of these interventions was designed as a structured, long-term programme implemented by statutory health insurance and conducted and evaluated as a randomized controlled trial. Enhancement of the understanding of HRQoL in diabetes and related risk factors is of great importance. We therefore studied HRQoL in patients with type 2 diabetes participating in the Austrian DMP “Therapie-Aktiv”.

## Methods

### Study aim

The aim of this study was to explore the impact of the Austrian DMP “Therapie-Aktiv” on HRQoL using the individual EQ-5D VAS and EQ-5D index in patients with type 2 diabetes. A multilevel model was used to identify variables that influence EQ-VAS and to assess its variation between patients, GPs and districts including a sex-specific analysis.

### Study design

This work was based on a pragmatic cluster-randomized controlled trial of the Austrian DMP “Therapie-Aktiv” in the province of Salzburg, Austria. The trial was approved by the ethics committee of Salzburg and registered with current controlled trials Ltd. (ISRCTN27414162) on July 12, 2007. The methodology was described in our study protocol [21] and the main findings (significant improvement of process quality but no significant change in metabolic control after 1 year) have been published previously [15].

### Setting of the study and characteristics of participants

Participation in the study was offered to all 275 family physicians and specialists for internal medicine having a contract with the Salzburg statutory health insurance. All patients (> 18 years) with type 2 diabetes that fulfilled the WHO/ADA-criteria for diabetes diagnosis were eligible to participate in the study. The recruitment period was July 1st to October 31st of 2007. Exclusion criteria were dementia/psychiatric illness with inability to participate or to give informed consent, or known major consuming illness (i.e. advanced cancer).

### Intervention

The Austrian DMP for type 2 diabetes “Therapie-Aktiv” [22] was developed in 2004 by the Austrian statutory health insurance. The programme aims to prevent diabetes complications and to improve quality of life through: (1) prevention and health promotion; (2) structured diagnosis and medical treatment; (3) considering patients’ overall cardiovascular risk; and (4) stronger patient involvement. Patients must sign a participant consent form to be enrolled in the programme. The attending GPs administer the patient informed consent. Patients participate in an education course on type 2 diabetes where they learn how to manage their disease. They receive a “patient’s booklet” which deals with topics such as healthy lifestyle (e.g. nutrition, exercise), blood glucose management, diabetes medication, and preventable long term complications. “Therapie-Aktiv” includes five main components:

1. Physician training consisting of an obligatory 10-h face to face course. This training was designed by the Austrian Diabetes Association, the Austrian Medical College and the Austrian Society for General Practice. It comprised an update in diabetes care, current guidelines of the Austrian Diabetes Association and practice management training;
2. Patient education consisting of 9 h face to face courses in small groups. The patient education was organized by the Working Group for Preventive Medicine Salzburg (Arbeitskreis Vorsorgemedizin Salzburg) using the “Düsseldorfer Modell” curriculum [23];
3. Quarterly patient-physician encounters of 15–20 min duration to discuss the results of laboratory tests (HbA1c, eGFR, microalbumine) and physical examination (BMI, blood pressure, foot examination, neurological examination) and determine treatment goals for the next quarter year, including standardised documentation of physical examination, laboratory findings and diabetes complications in a DMP case report form once a year [22];
4. Structured interdisciplinary care according to the guidelines of the Austrian Diabetes Association [24]; and
5. Agreement on therapeutic goals in a shared patient–physician decision-making process. These agreements are signed by the patient and physician every third month.

In the control group, physicians performed usual care.

### Data collection

Baseline and follow-up data were collected by the responsible GP. Standardised documentation of physical examination and laboratory findings in a DMP-form was performed once a year. The DMP form was administered in both study groups. Patients’ education and occupational status, smoking status, living situation, nationality, macrovascular diabetic complications (i.e. myocardial infarction, PTCA/stenting, coronary bypass, stroke, carotid surgery, amputation/gangrene, peripheral artery bypass or PTA), and self-rated HRQoL (EQ-5D-3L) were assessed by a standardised questionnaire. Follow-up data were collected after 12 months intervention.

### Outcomes and statistical analysis

We used the EQ-5D-3L to evaluate the self-rated HRQoL [25, 26]. The EQ-5D-3L consists of two parts: the EQ-5D descriptive system and the EQ visual analogue scale (EQ-VAS). The EQ-5D-3L descriptive system comprises five dimensions: mobility, self-care, usual activities, pain/discomfort and anxiety/depression. Each dimension has three levels: no problems, some problems, extreme problems. The EQ-index score is calculated based on the results of the five dimensions by using a scoring algorithm. The EQ-VAS records the respondent’s self-rated health on a vertical thermometer-like scale (0–100) where the endpoints are labelled worst imaginable health state’ and ‘best imaginable health state’. Following the user’s guide of the EuroQol Group [26], ambiguous scores were treated as missing. Results were reported according to recent guidelines [27]. For comparison of rates, we used Fisher’s Exact Test; for continuous variables we used the independent T-Test or Welch-Test. To assess changes over the study period of 1 year and in order to find factors that influence HRQoL outcomes, we analysed changes of EQ-VAS and EQ-index within and between groups, using repeated measures general linear model. Patients with at least 300 days of study duration and with two valid EQ-VAS scores (baseline and follow-up) were included in the analysis. In order to evaluate the final HRQoL we identified variables that explain some of the variation in final EQ-VAS and EQ-index as well as to assess their variation at different levels within a multilevel framework. We further incorporated sex-specific variables and examined the effect of living situation (living alone or not), education and occupational status (fulltime work or not), nationality (Austrian or not), any manifestation of coronary heart disease (myocardial infarction and/or PTCA/stenting and/or coronary bypass), any macrovascular diabetic complication (myocardial infarction and/or PTCA/stenting and/or coronary bypass and/or stroke and/or carotid surgery and/or amputation/gangrene and/or peripheral artery bypass or PTA) as they are known to potentially influence HRQoL within a diabetic population. We analysed educational level as low (no school leaving examination) or high (school leaving examination or higher). A full guideline adherence treatment covered four aspects: patient education, diagnostic measures (i.e. regular HbA1c-checks), ophthalmological examination, and foot examinations.

The multilevel modeling takes three levels into account: patient-level (sex, age, nationality, education, occupational, family and smoking status, study length), GP-level (intervention or control group), and district-level (“rural”, “urban” or “mixed”). We started modeling patients’ HRQoL by separating all three sources of variation. Hence, our multilevel linear model can be written as follows: $y_{ijk} \, = \,\beta_{0ijk} \, + \,\varvec{\beta X}_{ijk} \, + \,\varvec{\gamma Y}_{jk} \, + \,\varvec{\delta Z}_{k}$, where *y*_*ijk*_ represents the dependent variable, i.e. final EQ-VAS or final EQ-index, that is a function of explanatory variables on patient-level (**X**), GP-level (**Y**) and district-level (**Z**) with corresponding coefficients **β**, **γ** and **δ**, respectively. The overall error term *β*_*0ijk*_ can be decomposed into *β*_*0*_+ *v*_*0k*_+ *u*_*0jk*_+ *e*_*0ijk*_, where *v*_*0k*_ is the random error term for the kth district, *u*_*0jk*_ denotes the GP effect (of the jth GP within the kth district) and *e*_*0ijk*_ denotes the patient residual (error term of ith patient treated by the jth GP within the kth district). We examined various models, continuously adding variables to explain the variation in the dependent variable, starting with an empty model, including only the random intercept. We analysed random effects parameters in order to disentangle variation of the final EQ-VAS. For analyses we used IBM© SPSS© Statistics Version 19 and MLWiN 2.13 [28].

## Results

### Characteristics of the study population

**Fig. 1:**
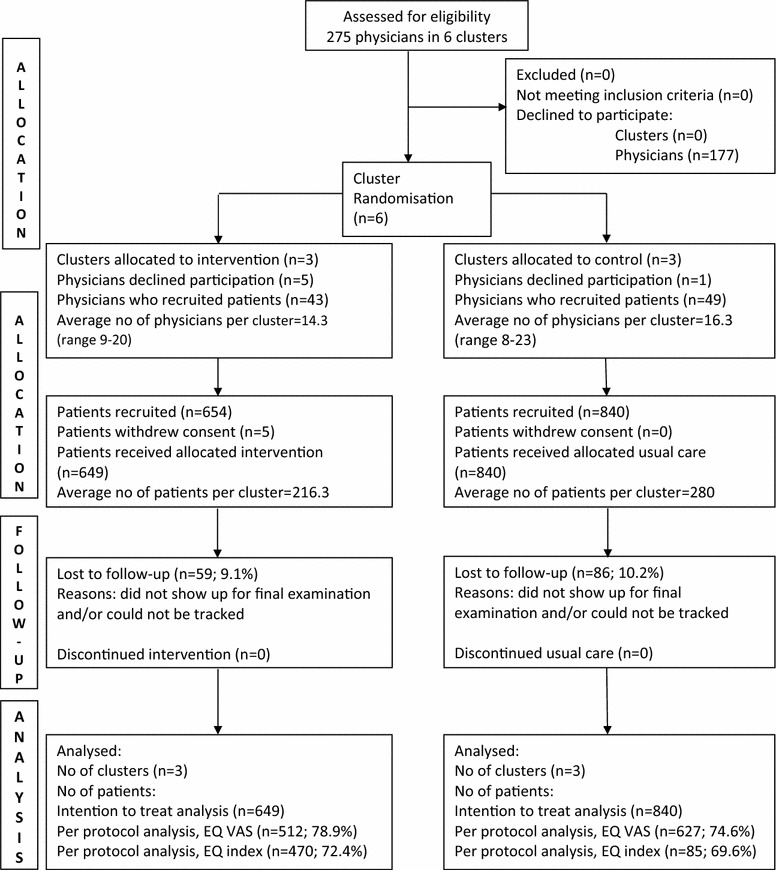
Consort chart

Ninety-two (33.5%) physicians participated and recruited 1489 patients in the province of Salzburg, Austria. Three hundred fifty patients were excluded from analysis (328 patients did not report two valid EQ-VAS scores and further 22 patients had a study period of less than 300 days). No significant differences were shown in baseline characteristics between included (n = 1139) and excluded (n = 350) patients. The mean length of the study period was 398 days (SD 42). EQ-VAS was available in 512 (78.9%) participants in the intervention group and in 627 (74.6%) controls (Fig. 1). There were no relevant demographic differences between intervention and control group at baseline (Additional files 1 and 2). There were significant differences in baseline data disaggregated by sex (Table 1). In various subgroups there were significant differences in EQ-VAS (Table 2), EQ-index, and EQ five dimensions (Additional files 3, 4, 5).

**Table 1:** Baseline characteristics disaggregated by sex (per protocol analysis population)

	Number of participants	Total	Female	Male	
	Total/ female	Proportion in %	Proportion in %	Proportion in %	p-value^a^
Intervention group (%)	1139/543	45.0	45.9	44.1	0.592
Austrian (%)	1136/540	94.5	95.6	93.6	0.191
Living alone (%)	1116/532	22.0	30.6	14.0	< 0.001
Higher education (school leaving examination or higher) (%)	1128/538	8.5	5.4	11.4	< 0.001
Working fulltime (%)	1136/541	14.0	7.0	20.3	< 0.001
Smoker (%)	1139/543	13.8	10.3	16.9	0.001
Any manifestation of coronary heart disease (%)^c^	1139/543	14.7	10.1	18.8	< 0.001
Any macrovasculardiabetic complication (%)^d^	1139/543	24.1	19.5	28.2	0.001

	Total/ female	Mean ± SD	Mean ± SD	Mean ± SD	p-value^b^
Age (mean years ± SD)	1139/543	65.42 ± 10.28	66.89 ± 10.56	64.07 ± 9.84	< 0.001
Duration of diabetes (mean years ± SD)	1089/519	7.13 ± 6.76	7.34 ± 7.08	6.95 ± 6.46	0.349
HbA1c (% ± SD)	1139/543	7.37 ± 1.38	7.39 ± 1.31	7.35 ± 1.44	0.614
Creatinine (µmol/l ± SD)	1138/542	0.96 ± 0.36	0.87 ± 0.31	1.04 ± 0.39	< 0.001
Triglycerides (mmol/l ± SD)	1139/543	181.40 ± 155.71	168.66 ± 117.97	193.00 ± 182.78	0.143
Cholesterol (mmol/l ± SD)	1139/543	195.75 ± 42.95	204.02 ± 43.62	188.21 ± 40.83	< 0.001
HDL (mmol/l ± SD)	1137/542	51.25 ± 14.35	55.71 ± 14.62	47.18 ± 12.83	< 0.001
LDL (mmol/l ± SD)	1091/530	110.03 ± 35.75	115.45 ± 37.34	104.91 ± 33.42	< 0.001
Systolic blood pressure (mmHg ± SD)	1139/543	139.41 ± 17.78	140.34 ± 18.16	138.56 ± 17.39	0.092
Diastolic blood pressure (mmHg ± SD)	1139/543	82.22 ± 10.61	82.10 ± 10.87	82.34 ± 10.39	0.700
BMI (kg/m^2^)	1139/543	29.99 ± 4.91	30.20 ± 5.30	29.81 ± 4.52	0.301
EQ-VAS	1139/543	71.10 ± 18.01	69.24 ± 18.81	72.80 ± 17.09	0.002
EQ-index	1098/519	0.88 ± 0.17	0.86 ± 0.19	0.90 ± 0.16	< 0.001

^a^Fisher’s exact test or Chi Square-Test, respectively

^b^Independent T Test or Mann–Whitney-Test, respectively

^c^Myocardial infarction and/or PTCA/stenting and/or coronary bypass

^d^Myocardial infarction and/or PTCA/stenting and/or coronary bypass and/or stroke and/or carotid surgery and/or amputation/gangrene and/or peripheral artery bypass or PTA

**Table 2:** EQ-VAS at baseline, values for selected subgroups (intention-to-treat analysis population)

Subgroup 1/subgroup 2	No of participants, subgroup 1/subgroup 2	EQ-VAS, mean [CI], subgroup 1	EQ-VAS, mean [CI], subgroup 2	p value^a^
Overall study population	1464	70.1 [69.1;71.0]		–
Intervention/control	639/825	69.4 [68.0;70.8]	70.6 [69.3;71.8]	0.231
Female/male	700/764	68.4 [67.0;69.8]	71.6 [70.3;72.8]	0.001
No manifestation of coronary heart disease^b^/ any manifestation of coronary heart disease	1253/211	70.6 [69.6;71.6]	66.6 [64.1;69.2]	0.003
No macrovascular diabetic complication^c^/ any macrovascular diabetic complication	1108/356	71.4 [70.3;72.4]	66.0 [64.1;68.0]	< 0.001
Living with a partner/ living alone	1111/318	71.0 [69.9;72.0]	66.8 [64.8;68.8]	< 0.001
Non Austrian/Austrian	80/1377	65.1 [60.6;69.7]	70.3 [69.4;71.3]	0.029
No higher education/ higher education	1327/117	69.9 [68.9;70.9]	72.1 [68.9;75.4]	0.202
Working fulltime/ not working fulltime	1255/202	69.7 [68.7;70.7]	72.4 [69.9;74.9]	0.050

^a^Independent T Test or Welch-Test, respectively

^b^Myocardial infarction and/or PTCA/stenting and/or coronary bypass

^c^Myocardial infarction and/or PTCA/stenting and/or coronary bypass and/or stroke and/or carotid surgery and/or amputation/gangrene and/or peripheral artery bypass or PTA

### Outcomes—univariate analysis

In univariate analysis, the EQ-VAS improved within both groups. It increased by 2.19 points (p = 0.005) in the intervention group and by 1.18 points (p = 0.094) in controls. The mean difference (1.00) between intervention and control group was not significant (p = 0.339). In the intervention group the improvement was stronger in women (2.46, p = 0.036) compared to men (1.92, p = 0.063). Significant differences in EQ-VAS scores were found in various subgroups within the intervention group (Table 3). The EQ-index score hardly changed during the study period, so no multilevel analysis was performed for the EQ-index. The mean difference within the intervention group was 0.00 (p = 0.612) (Additional file 6).

**Table 3:** EQ-VAS in the intervention group at follow-up, various subgroups disaggregated by sex

	Number of participants	EQ-VAS, mean difference ± SD		p value		
Total	512	2.19 ± 17.55		0.005		
Subgroups	Female/male	Female	Male	p value^a^	p value^b^	p value^c^
Female/male	249/263	2.46 ± 18.46	1.92 ± 16.68	0.005	0.729	< 0.001
Non Austrian	10/15	1.50 ± 24.50	− 0.33 ± 27.12	0.914	0.865	0.490
Austrian	239/248	2.50 ± 18.23	2.06 ± 15.91	0.003	0.776	0.029
Living with a partner	159/223	2.48 ± 17.95	2.25 ± 16.59	0.008	0.897	0.002
Living alone	85/35	2.81 ± 19.77	− 0.71 ± 17.94	0.589	0.364	0.425
High education (school leaving examination or higher)	17/32	3.53 ± 18.18	3.47 ± 17.57	0.196	0.991	0.073
No higher education	229/229	2.28 ± 18.61	1.77 ± 16.64	0.014	0.755	0.002
Not working fulltime	227/211	2.30 ± 18.51	2.73 ± 16.15	0.003	0.796	0.001
Working fulltime	21/52	3.81 ± 18.63	− 1.37 ± 18.47	0.611	0.283	0.046
Non-smoker	222/221	2.18 ± 18.09	2.53 ± 16.28	0.004	0.831	< 0.001
Current smoker	27/42	4.74 ± 21.52	− 1.29 ± 18.54	0.481	0.220	0.122
No manifestation of coronary heart disease^d^	219/216	2.77 ± 18.23	1.69 ± 17.51	0.010	0.528	< 0.001
Any manifestation of coronary heart disease^d^	30/47	0.23 ± 20.22	3.02 ± 12.26	0.381	0.453	0.410
No macrovascular diabetic complication^e^	189/192	3.06 ± 17.39	2.11 ± 17.16	0.004	0.592	0.001
Any macrovascular diabetic complication^e^	60/71	0.58 ± 21.53	1.42 ± 15.41	0.537	0.796	0.022
Non guideline adherence treatment^f^	130/134	2.22 ± 17.34	1.21 ± 17.44	0.110	0.636	0.004
Full guideline adherence treatment^f^	119/129	2.72 ± 19.68	2.67 ± 15.89	0.018	0.980	0.012

^a^Within-subject factor (time)

^b^Within-subject factor (time*sex)

^c^Between subject factors (sex)

^d^Myocardial infarction and/or PTCA/stenting and/or coronary bypass

^e^Myocardial infarction and/or PTCA/stenting and/or coronary bypass and/or stroke and/or carotid surgery and/or amputation/gangrene and/or peripheral artery bypass or PTA

^f^A full guideline adherence treatment covered four aspects: patient education; diagnostic measures (i.e. regular HbA1c-checks); ophthalmological and foot examinations

### Outcomes—multilevel analysis

Most of the variance was found on patient-level (approximately 95%). The GP-level (intervention or control group) and district-level (“rural”, “urban” or “mixed”) had only a modest (5%) or no impact (0%). Due to the negligible impact of district-level and the restricted number of districts (n = 6), we decided to continue with two levels, GP-level and patient-level. The results of the empty model (“2-level null model”) and the baseline-adjusted model for two levels (“baseline-adjusted”) are presented in Table 4. Adding the baseline score accounted for 27.0% of the within GP-level variability in the EQ-VAS final score, and 30.9% of the variation in means across GPs could be attributed to differences in the baseline score of patients nested in these GP-surgeries. We continued adding variables in the fixed part of the model to examine variation in final EQ-VAS. First, we included patient characteristics (sex, age, nationality, education, occupational, family and smoking status, and study duration) as well as group association. The parameter estimate for study duration was negligible (0.01, SE 0.01), hence we present the results of this model (“socio-economic”) without adjusting for study length. Age, living alone, female sex and baseline EQ-VAS have a significant and negative impact on final EQ-VAS. The enrolment in the DMP had no significant impact on final EQ-VAS (p = 0.646). Adding diabetes associated parameters, led to a slight decrease of unexplained variance. In patients with any macrovascular diabetic complication and longer diabetes duration the final EQ-VAS was significantly reduced (p = 0.021 and p = 0.002, respectively). Any manifestation of coronary heart disease had no significant impact on the final EQ-VAS. Subsequently, we adjusted for significant baseline differences (cholesterol, BMI) and guideline adherence. The addition of further variables consecutively reduced the amount of unexplained variance. However, the allocation of variation did not change much. In all models, the intraclass correlation coefficient on GP-level amounted to approximately 5%. Since baseline EQ-VAS was significantly related to the dependent variable, we checked whether the slope varied randomly across GPs. However, adding the random slope hardly changed parameter estimates and slope variance was not significant. Composition effects did not exist for EQ-VAS. In the final model, the final EQ-VAS score in the intervention group was on average 1.17 points higher compared to controls (while retaining all variables unchanged), however not significant (p = 0.390).

**Table 4:** EQ-VAS, fixed and random part results of selected models, intervention- and control group at follow up (multilevel modeling)

	3-level null model	2-level null model	Baseline-adjusted model	Socio-economic model	Baseline and full guideline adherence treatment model	Final model
*Fixed part* ^a,b^						
Constant	72.52 (0.89)*	72.65 (0.74)*	35.67 (1.89)*	40.95 (4.29)*	48.17 (5.98)*	48.22 (6.01)*
*Patient characteristics*						
Sex (female/male)				− 2.23 (0.96)°	− 2.29 (0.99)°	− 2.30 (0.99)°
Age (mean years ± SD)				− 0.13 (0.06)°	− 0.12 (0.06)°	− 0.12 (0.06)°
Austrian (yes/no)				− 2.19 (2.08)	− 2.78 (2.10)	− 2.68 (2.10)
Living alone (yes/no)				2.36 (1.15)°	2.48 (1.17)°	2.36 (1.17)°
Higher education (school leaving examination or higher)				0.41 (1.66)	− 0.27 (1.69)	− 0.39 (1.70)
Working fulltime (yes/no)				2.82 (1.58)	3.51 (1.62)°	3.44 (1.62)°
Smoker (yes/no)				0.78 (1.39)	1.34 (1.42)	1.20 (1.42)
Any macrovascular diabetic complication^c^					2.25 (1.09)°	2.23 (1.09)°
Duration of diabetes (year)					− 0.23 (0.07)*	− 0.22 (0.07)*
*Baseline values*						
EQ-VAS			0.52 (0.03)*	0.50 (0.03)*	0.48 (0.03)*	0.48 (0.03)*
Body mass index (kg/cm^2^)					− 0.25 (0.10)°	− 0.25 (0.10)°
*DMP associated variables*						
Group assignment (Intervention or control group)				0.61 (1.27)	1.14 (1.35)	1.17 (1.36)
Full guideline adherence treatment^d^					0.90 (1.12)	1.03 (1.13)
*District type*						
Rural						1.46 (1.47)
Urban						− 1.04 (1.67)
*Random Part* ^b^						
District-level (“rural”, “urban” or “mixed”)	1.56 (3.40)					
GP-level (intervention or control group)	15.73 (6.42)	17.36 (6.10)	11.99 (4.25)	12.10 (4.37)	11.72 (4.44)	10.51 (4.27)
Patient characteristics-level (sex, age, nationality, education, occupational, family and smoking status, study length)	307.22 (13.12)	307.12 (13.12)	224.12 (9.64)	221.40 (9.69)	218.29 (9.78)	218.47 (9.79)
Intraclass correlation coefficient, district level	0.0048					
Intraclass correlation coefficient, GP level	0.0485	0.0535	0.0508	0.0518	0.0509	0.0459
− 2 Log-Likelihood	9962.12	9962.42	9438.57	9127.70	8706.66	8704.46
Number of districts	6					
Number of GPs	79	79	79	79	74	74
Number of patients	1139	1139	1139	1103	1054	1054

Interpretation based on the socio-economic model						
Model	Interpretation					
Socio-economic model	The following baseline variables have a significantly negative impact on the final EQ-VAS: low baseline EQ-VAS score, female sex, higher age, and living alone. In the socio-economic model the baseline EQ-VAS variable was positively (0.50) and the age variable negatively associated (− 0.13). These numbers should be interpreted as follows: a high EQ-VAS score at baseline and younger age impacts positively on final EQ-VAS The variable sex was negatively (− 2.23) associated. This means that the final EQ-VAS score is lower in woman compared to men. Participants living with a partner (variable living alone, positively associated [2.36]) have a higher final EQ-VAS score compared to those living alone					

^a^For the fixed part results ° and * represent significant results at the 5 and 1% level, respectively

^b^Standard errors are in parenthesis

^c^Myocardial infarction and/or PTCA/stenting and/or coronary bypass and/or stroke and/or carotid surgery and/or amputation/gangrene and/or peripheral artery bypass or PTA

^d^A full guideline adherence treatment covered four aspects: patient education; diagnostic measures (i.e. regular HbA1c-checks); ophthalmological and foot examinations

The final model explains 28.9% (EQ-VAS) of the total variance. Our multilevel analysis demonstrates that approximately 95% of the residual variation in final EQ-VAS is attributable to patient-level, with baseline EQ-VAS showing to be positively associated with the final score. Most of the unexplained variance can be found on patient-level (approximately 95%) and less on GP-level (approximately 5%).

## Discussion

### Main findings

This is the first study analysing the impact of a DMP implemented by statutory public health insurance on HRQoL in patients with type 2 diabetes in a cluster randomised controlled trial. A further aim was to evaluate HRQoL within a multilevel framework as a result of the cluster-design. A special focus was put on a sex-specific analysis.

The EQ-VAS significantly increased within the intervention group and the improvement was stronger in women than in men. This effect was not present when comparing mean differences between the intervention and control group. This may at least partly be due to a Hawthorne-effect in the control group mitigating the success of the intervention, but even when taking this into account the effect of the DMP on HRQoL is small and hardly of clinical relevance (see discussion of minimal important difference of EQ-VAS below). As the DMP also did not have a significant effect on metabolic control, and the observation period of one year was too short to detect any effect regarding clinical endpoints, a large impact on HRQoL of this DMP-programme directed mainly at the improvement of process quality may not be expected. In our multilevel model we demonstrated that sex, age, family and work circumstances, any macrovascular diabetic complication, duration of diabetes, baseline body mass index and baseline EQ-VAS significantly influence EQ-VAS at follow-up, while DMP “Therapie-Aktiv” does not. Our results confirm that effects are easily overestimated in the absence of a control group, as also shown in a previous study [29]. While changes showed to be significant in many subpopulations of the intervention group, this effect disappeared after putting it in relation to the control group. Significant differences were found in the baseline data disaggregated by sex (Table 1) and in various subgroups (Table 2). For the EQ-VAS, men reported to have better HRQoL in comparison to women. However, some of the differences might be affected by social desirability bias: men are probably less likely to admit deficits in quality of life than women. In pre-post analysis, DMP patients with higher education exhibited the greatest improvement, whereas higher educated controls showed the worst development among all subgroups. Considering these results, the design and development of effective DMPs remains an important—yet open—issue [17, 30], and it may be concluded that “Therapie aktiv” needs to be intensified and targeted at outcome quality to be effective.

When analysing our results it also has to be kept in mind that although a multilevel model was used to assess final EQ-VAS variation between patients, GPs and districts, almost all variation (approximately 95%) in final EQ-VAS was attributable to variables at the patient-level (e.g. age, sex, family and occupational status, duration of diabetes, baseline BMI and baseline EQ-VAS).

### Strengths and limitations

Our trial is characterised by a high level of internal validity. Minor limitations regarding its external validity exist. As pointed out previously [15], due to a volunteer-based enrolment strategy, GPs might have recruited “healthier” and more compliant patients. Hence, it is questionable whether our sample accurately represents the population from which it was recruited. However, this might also reflect healthier patient characteristics in primary care settings.

We are aware of the limitations of the EuroQol tool (EQ-5D-3L) and of its use to evaluate HRQoL. Some researchers point out that the EQ-5D-3L rather measures quality of health [6, 31]; while others have shown that it is an appropriate tool to evaluate HRQoL among others in patients with type 2 diabetes [32–34]. Furthermore, the restricted number of levels of the EQ-5D can be criticised. However in 2007, when the Austrian DMP “Therapie-Aktiv” was rolled out, the latest version of the EQ-5D (five levels) [26], was not yet available.

There is a lack of data regarding minimal important difference on EQ-VAS in diabetes patients. Minimal important difference estimates on EQ-5D VAS ranged from 8 to 12 point scores in cancer patients [35]. It is questionable if an improvement of 2.19 points in our diabetes population is clinically relevant. Twelve months follow-up might have been too short in order to demonstrate greater improvements and moreover we know that the HRQoL decreases with disease progression and complications [6, 7].

We did not assess psychosocial comorbidities like depression which can be a significant confounder regarding HRQoL, because it was not part of the regular DMP-assessment of the Austrian “Therapie aktiv”. We assume, though, that these comorbidities are evenly distributed between intervention and control group due to randomization and do not affect the main result of our study (i.e. lack of a significant difference regarding HRQoL between intervention and control).

We used a multilevel model to assess final EQ-VAS variation between patients, GPs and districts taking into account possible predictive factors. Most of the variation (approx. 95%) could be observed on patient-level, whereas only little was attributable to GP (approx. 5%) or district (< 0.1%) level. In our data, only a restricted number of GP-level characteristics was available. However, the variation on GP-level was small. Hence, adding additional variables on GP-level or district-level would not have changed the overall picture. The choice of variables used in subgroup analysis and multilevel modeling was based on our baseline-analysis, and on previous studies [5, 7–12] that identified age, sex, family and work circumstances, level of education, residential area, ethnicity, and any macrovascular diabetic complication, as possible predictive factors for HRQoL. We are well aware of the problems and limitations of exploratory subgroup analysis/multiple testing and the loss of power involved. However, we wanted to present some of the material as a possible basis for further research and for future development of DMPs.

### Comparison with existing literature

A wide variety of educational, self-management, and structured care interventions with the aim to improve HRQoL in diabetic populations exists [16–18, 36–45]. Still, high quality randomized controlled trials are rare and little is known on the impact of DMPs on HRQoL [46]. Some studies demonstrated improvement in HRQoL [16–18, 36, 40, 43] and others did not [37–39, 44, 45]. Commonly, methodological and statistical heterogeneity exist (e.g. poor or missing randomisation, high dropout rates, pre-post comparisons, small sample size). Additionally, differences are present in composition and complexity of interventions. Duration of follow up and outcome measures are heterogeneous and hence difficult to compare. The effects seem to be largely dependent on the individual programme and its particular design [46].

### Implications for practice and future research

A major drawback of disease management programmes is that they probably do not reach a considerable number of higher risk patients [47]. Our results suggest that further development of DMPs in the sense of especially focusing on patients’ needs is necessary. Future research should focus on how to recruit high risk patients and how to promote patient-centred self-management and adherence to such a programme. There is a trend in Europe towards patient-centred self-management of diabetes in the primary care context. The professional role of diabetes specialist nurses, the need for multidisciplinary approaches and a focus on patient education emerge as fundamental principles in the design of relevant programmes [48], which should be strengthened in DMPs.

Since the Austrian DMP “Therapie-Aktiv” apparently has no significant impact on HRQoL after one year of intervention, we propose twofold: on the one hand, the intervention has to be intensified in terms of increasing patient empowerment and self-management. A promising and feasible approach to increase patient empowerment and adherence of patients enrolled in the DMP is initiating a peer support programme with regular physical activity, nutrition, psychological counselling, and diabetes relevant group discussions [49, 50]. Secondly, future programmes should try to better incorporate the needs of certain subpopulations. Our data show clearly that patients who are living alone, are of non-Austrian nationality, and are suffering from any macrovascular diabetic complication start off with lower EQ-VAS scores (up to five points below the average). This may also apply to other subgroups like patients with specific comorbidities (e.g. depression) not addressed in our study. Hence attention of future DMPs should especially focus on these subgroups.

## Conclusion

In this cluster randomised controlled trial, the Austrian DMP “Therapie-Aktiv” showed no significant impact on EQ-VAS or EQ-index when compared to usual care although the EQ-VAS increased significantly within the intervention group. Our multilevel model to assess final EQ-VAS variation by patient-level, GPs’ group assignment and district-level demonstrated that age, sex, family and occupational status, duration of diabetes, baseline BMI and baseline EQ-VAS have a significant impact on EQ-VAS at follow-up. Almost all variation was attributable to patient-level. Future research and development of DMPs should focus on subgroups with lower HRQoL, patient needs, and predictors of HRQoL in order to maintain or improve HRQoL.

## Key message

- The EQ-VAS increased significantly within the intervention group and the improvement was stronger in women compared to men. This effect was not present in group comparison.
- The multilevel model demonstrated that sex, age, family and work circumstances, existence of any macrovascular diabetic complication, duration of diabetes, baseline body mass index and baseline EQ-VAS significantly influence EQ-VAS at follow-up.
- Future evaluations of the impact of DMPs on HRQoL in type 2 diabetes should incorporate predictors of HRQoL such as sex, age, socioeconomic status, coronary heart disease, and macrovascular diabetes complications in their analysis.

## Additional files

**Additional file 1.** Baseline data (intention-to-treat analysis population).

**Additional file 2.** Baseline data disaggregated by sex (intention-to-treat analysis population).

**Additional file 3.** Baseline data, EQ-VAS subgroup analysis (per protocol analysis population).

**Additional file 4.** Baseline data, EQ-index subgroup analysis.

**Additional file 5.** Baseline data EQ-5 dimensions disaggregated by sex.

**Additional file 6.** EQ-index in the intervention group at follow-up, various subgroups disaggregated by sex.

## Abbreviations

BMI: body mass index
DMP: disease management programme
GP: general practitioner
HRQoL: health related quality of life
RCT: randomized controlled trial
SGKK: Salzburger Gebietskrankenkasse (in English, the statutory public health insurance of the province of Salzburg)
