# Supplementary material for: The impact of a disease management programme for type 2 diabetes on health-related quality of life: multilevel analysis of a cluster-randomised controlled trial
Source: Diabetol Metab Syndr. 2018 Apr 10;10:28. doi: 10.1186/s13098-018-0330-9 (PMC5892002; doi:10.1186/s13098-018-0330-9)
Supplement: Supplementary file 1 — Additional file 1. Baseline data (intention-to-treat analysis population). [file 13098_2018_330_MOESM1_ESM.docx]

Additional file 1 Baseline data (intention-to-treat analysis population)

|  | **Intervention** | **Control** | **p-value** |
| --- | --- | --- | --- |
| Total number of patients | 649 | 840 |  |
| Number of GPs | 43 | 49 |  |
| Age (mean years ± SD) | 65.5 ± 10.3 | 65.6 ± 10.3 | 0.841^2^ |
| Female (%) | 49.0 | 46.9 | 0.433^1^ |
| Austrian (%) | 94.6 | 93.2 | 0.227^1^ |
| Living alone (%) | 23.6 | 20.1 | 0.232^1^ |
| Higher education (%) | 8.9 | 7.1 | 0.441^1^ |
| Working fulltime (%) | 13.6 | 13.8 | 0.202^1^ |
| Smoker (%) | 12.9 | 13.8 | 0.690^1^ |
| Any manifestation of coronary heart disease (%)^3^ | 13.3 | 15.6 | 0.209^1^ |
| Any macrovascular diabetic complication (%)^4^ | 21.9 | 26.3 | 0.052^1^ |
| Duration of diabetes (years ± SD) | 7.1 ± 6.5 | 6.9 ± 6.7 | 0.718^2^ |
| HbA1c (% ± SD) | 7.5 ± 1.5 | 7.3 ± 1.3 | 0.098^2^ |
| Creatinine (µmol/l ± SD) | 84.9 ± 35.4 | 84.9 ± 35.4 | 0.922^2^ |
| Triglycerides (mmol/l ± SD) | 2.1 ± 1.8 | 2.0 ± 1.7 | 0.121^2^ |
| Cholesterol (mmol/l ± SD) | 5.2 ± 1.1 | 5.0 ± 1.1 | 0.023^2^ |
| HDL (mmol/l ± SD) | 1.3 ± 0.4 | 1.3 ± 0.4 | 0.602^2^ |
| LDL (mmol/l ± SD) | 2.9 ± 1.0 | 2.9 ± 0.9 | 0.779^2^ |
| Systolic blood pressure  (mmHg ± SD) | 140.6 ± 18.9 | 139.1 ± 17.0 | 0.121^2^ |
| Diastolic blood pressure  (mmHg ± SD) | 82.5 ± 11.2 | 82.1 ± 10.1 | 0.413^2^ |
| BMI (kg/m²) | 30.4 ± 5.1 | 29.7 ± 4.9 | 0.012^2^ |

^1^ Fisher’s exact test.

^2^ Independent T-test.

^3^ Myocardial infarction and/or PTCA/stenting and/or coronary bypass

^4^ Myocardial infarction and/or PTCA/stenting and/or coronary bypass and/or stroke and/or carotid surgery and/or amputation/gangrene and/or peripheral artery bypass or PTA
