# Supplementary material for: The impact of a disease management programme for type 2 diabetes on health-related quality of life: multilevel analysis of a cluster-randomised controlled trial
Source: Diabetol Metab Syndr. 2018 Apr 10;10:28. doi: 10.1186/s13098-018-0330-9 (PMC5892002; doi:10.1186/s13098-018-0330-9)
Supplement: Supplementary file 2 — Additional file 2. Baseline data disaggregated by sex (intention-to-treat analysis population). [file 13098_2018_330_MOESM2_ESM.docx]

Additional file 2 Baseline data disaggregated by sex (intention-to-treat analysis population)

|  | **Number of participants** | **Total** | **Female** | **Male** |  |
| --- | --- | --- | --- | --- | --- |
|  | **Total/**  **female** | **Proportion in %** | **Proportion in %** | **Proportion in %** | **p-value**^1^ |
| Intervention group (%) | 1489/712 | 43.6 | 44.7 | 42.6 | 0.433 |
| Austrian (%) | 1482/707 | 94.3 | 95.2 | 93.4 | 0.148 |
| Living alone (%) | 1452/691 | 22.2 | 31.5 | 13.7 | <0.001 |
| Higher education (school leaving examination or higher) (%) | 1465/696 | 8.1 | 5.0 | 10.8 | <0.001 |
| Working fulltime (%) | 1481/707 | 13.8 | 6.9 | 20.0 | <0.001 |
| Smoker (%) | 1489/712 | 14.3 | 10.8 | 17.5 | <0.001 |
| Any manifestation of coronary heart disease (%)^3^ | 1489/712 | 14.6 | 10.4 | 18.4 | <0.001 |
| Any macrovascular diabetic complication (%)^4^ | 1489/712 | 24.4 | 20.1 | 28.3 | <0.001 |
|  | **Total/**  **female** | **Mean ± SD** | **Mean ± SD** | **Mean ± SD** | **p-value^2^** |
| Age (mean years ± SD) | 1489/712 | 65.58 ± 10.35 | 67.09 ± 10.54 | 64.20 ± 9.98 | <0.001 |
| Duration of diabetes  (years ± SD) | 1296/624 | 7.01 ± 6.60 | 7.22 ± 7.00 | 6.82 ± 6.20 | 0.803 |
| HbA1c (% ± SD) | 1489/712 | 7.39 ± 1.41 | 7.40 ± 1.34 | 7.39 ± 1.47 | 0.861 |
| Creatinine (µmol/l ± SD) | 1485/709 | 0.96 ± 0.37 | 0.86 ± 0.29 | 1.05 ± 0.42 | <0.001 |
| Triglycerides (mmol/l ± SD) | 1489/712 | 182.08 ± 156.90 | 168.40 ± 111.32 | 194.62 ± 188.46 | 0.210 |
| Cholesterol (mmol/l ± SD) | 1489/712 | 196.46 ± 42.78 | 205.20 ± 42.58 | 188.45 ± 41.40 | <0.001 |
| HDL (mmol/l ± SD) | 1487/711 | 51.32 ± 14.58 | 55.74 ± 14.87 | 47.27 ± 13.07 | <0.001 |
| LDL (mmol/l ± SD) | 1429/695 | 110.97 ± 36.10 | 116.57 ± 36.95 | 105.67 ± 34.47 | <0.001 |
| Systolic blood pressure  (mmHg ± SD) | 1488/712 | 139.73 ± 17.87 | 140.45 ± 18.10 | 139.07 ± 17.65 | 0.138 |
| Diastolic blood pressure  (mmHg ± SD) | 1488/712 | 82.28 ± 10.57 | 82.02 ± 10.86 | 82.51 ± 10.31 | 0.370 |
| BMI (kg/m^2^) | 1489/712 | 30.00 ± 4.97 | 30.28 ± 5.39 | 29.74 ± 4.54 | 0.093 |
| EQ-VAS | 1464/700 | 70.05 ± 18.20 | 68.39 ± 18.60 | 71.58 ± 17.69 | 0.001 |
| EQ-index | 1411/671 | 0.86 ± 0.18 | 0.85 ± 0.19 | 0.90 ± 0.16 | <0.001 |

^1^ Fisher’s exact test or chi-square-test, respectively

^2^ Independent T-test or Mann-Whitney-test, respectively

^3^ Myocardial infarction and/or PTCA/stenting and/or coronary bypass

^4^ Myocardial infarction and/or PTCA/stenting and/or coronary bypass and/or stroke and/or carotid surgery and/or amputation/gangrene and/or peripheral artery bypass or PTA
