# Supplementary material for: The impact of a disease management programme for type 2 diabetes on health-related quality of life: multilevel analysis of a cluster-randomised controlled trial
Source: Diabetol Metab Syndr. 2018 Apr 10;10:28. doi: 10.1186/s13098-018-0330-9 (PMC5892002; doi:10.1186/s13098-018-0330-9)
Supplement: Supplementary file 3 — Additional file 3. Baseline data, EQ-VAS subgroup analysis (per protocol analysis population). [file 13098_2018_330_MOESM3_ESM.docx]

Additional file 3 Baseline data, EQ-VAS subgroup analysis (per protocol analysis population)

| **Subgroup 1/subgroup 2** | **Number of participants**  **(subgroup 1/ subgroup 2)** | **EQ-VAS, mean ± SD, subgroup 1** | **EQ-VAS, mean ± SD, subgroup 2** | **p-value^1^** |
| --- | --- | --- | --- | --- |
| EQ-VAS total population | 1139 | 71.10 ± 18.01 | | - |
| Female/male | 543/596 | 69.24 ± 18.81 | 72.80 ± 17.09 | 0.002 |
| Intervention/control | 512/627 | 70.54 ± 17.43 | 71.55 ± 18.48 | 0.347 |
| Non Austrian/Austrian | 62/1074 | 66.79 ± 20.99 | 71.31 ± 17.81 | 0.055 |
| Living with a partner | 871/245 | 72.08 ± 17.85 | 67.66 ± 18.47 | 0.001 |
| No higher education/higher education | 1032/96 | 71.03 ± 18.15 | 72.85 ± 16.47 | 0.342 |
| Working fulltime/not working fulltime | 977/159 | 70.70 ± 17.99 | 73.65 ± 18.02 | 0.055 |
| Non-smoker/current smoker | 982/157 | 71.54 ± 17.67 | 68.35 ± 19.89 | 0.095 |
| No macrovascular diabetic complication^2^/ Any macrovascular diabetic complication | 829/310 | 72.68 ± 17.76 | 66.88 ± 18.04 | <0.001 |
| Non guideline adherence treatment/full guideline adherence treatment^3^ | 802/337 | 71.25 ± 17.95 | 70.74 ± 18.18 | 0.666 |

^1^ Independent T-test or Welch-test, respectively

^2^ Myocardial infarction and/or PTCA/stenting and/or coronary bypass and/or stroke and/or carotid surgery and/or amputation/gangrene and/or peripheral artery bypass or PTA

^3^ A full guideline adherence treatment covered four aspects: patient education; diagnostic measures (i.e. regular HbA1c-checks); ophthalmological; and foot examinations
