# Supplementary material for: The impact of a disease management programme for type 2 diabetes on health-related quality of life: multilevel analysis of a cluster-randomised controlled trial
Source: Diabetol Metab Syndr. 2018 Apr 10;10:28. doi: 10.1186/s13098-018-0330-9 (PMC5892002; doi:10.1186/s13098-018-0330-9)
Supplement: Supplementary file 4 — Additional file 4. Baseline data, EQ-index subgroup analysis. [file 13098_2018_330_MOESM4_ESM.docx]

Additional file 4 Baseline data, EQ-index subgroup analysis

| **Subgroup 1/ subgroup 2** | **Number of participants,**  **(subgroup 1/**  **subgroup 2)** | **EQ-index,**  **mean [CI], subgroup 1** | **EQ-index,**  **mean [CI], subgroup 2** | **p-value^1^** |
| --- | --- | --- | --- | --- |
| Overall study population | 1411 | 0.88 [0.87;0.89] | | - |
| Intervention/control | 626/785 | 0.87 [0.86;0.89] | 0.88 [0.86;0.89] | 0.856 |
| Female/male | 671/740 | 0.85 [0.84;0.87] | 0.90 [0.88;0.91] | <0.001 |
| No manifestation of coronary heart disease^2^ / Any manifestation of coronary heart disease | 1203/208 | 0.88 [0.87;0.89] | 0.86 [0.83;0.89] | 0.208 |
| No macrovascular diabetic complication^3^/ Any macrovascular diabetic complication | 1063/348 | 0.89 [0.88;0.90] | 0.84 [0.82;0.86] | <0.001 |
| Living with a partner /living alone | 1074/308 | 0.88 [0.87;0.90] | 0.84 [0.82;0.87] | 0.001 |
| Non Austrian/Austrian | 80/1326 | 0.88 [0.84;0.92] | 0.88 [0.87;0.88] | 0.861 |
| No higher education/higher education | 1278/112 | 0.87 [0.86;0.88] | 0.90 [0.87;0.93] | 0.092 |
| Working fulltime/not working fulltime | 1205/201 | 0.87 [0.86;0.88] | 0.91 [0.89;0.94] | 0.001 |

^1^ Independent T-test or Welch-test, respectively

^2^ Myocardial infarction and/or PTCA/stenting and/or coronary bypass

^3^ Myocardial infarction and/or PTCA/stenting and/or coronary bypass and/or stroke and/or carotid surgery and/or amputation/gangrene and/or peripheral artery bypass or PTA
