# Supplementary material for: The impact of a disease management programme for type 2 diabetes on health-related quality of life: multilevel analysis of a cluster-randomised controlled trial
Source: Diabetol Metab Syndr. 2018 Apr 10;10:28. doi: 10.1186/s13098-018-0330-9 (PMC5892002; doi:10.1186/s13098-018-0330-9)
Supplement: Supplementary file 5 — Additional file 5. Baseline data EQ-5 dimensions disaggregated by sex. [file 13098_2018_330_MOESM5_ESM.docx]

Additional file 5 Baseline data EQ-5 dimensions disaggregated by sex

|  | **Number of participants (female/male)** | **Female: problems^1^** | | **Male: problems^1^** | | **p-value^2^** |
| --- | --- | --- | --- | --- | --- | --- |
|  |  | **no** | **extreme** | **no** | **extreme** |  |
| ***„mobility“*** | | | | | | |
| **Total** | **1126** | **no: 76.6%. some: 23.3%. extreme: 0.1%** | | | | **-** |
| Female/male (total) | 537/589 | 72.6% | 0.2% | 80.3% | 0.0% | 0.007 |
| Female/male (intervention group) | 246/261 | 74.8% | 0.4% | 82.4% | 0.0% | 0.079 |
| Female/male (control group) | 291/328 | 70.8% | 0.0% | 78.7% | 0.0% | 0.026 |
| ***„self-care“*** | | | | | | |
| **Total** | **1121** | **no: 94.7%. some: 4.8%. extreme: 0.4%** | | | | **-** |
| Female/male (total) | 533/588 | 92.9% | 0.4% | 96.4% | 0.5% | 0.015 |
| Female/male (intervention group) | 245/261 | 95.1% | 0.4% | 96.2% | 0.4% | 0.834 |
| Female/male (control group) | 288/327 | 91.0% | 0.3% | 96.6% | 0.6% | 0.005 |
| ***„usual activities“*** | | | | | | |
| **Total** | **1124** | **no: 85.6%. some: 13.3%. extreme: 1.1%** | | | | **-** |
| Female/male (total) | 535/589 | 81.9% | 1.1% | 89.0% | 1.0% | 0.003 |
| Female/male (intervention group) | 244/261 | 84.0% | 1.2% | 89.3% | 0.4% | 0.173 |
| Female/male (control group) | 291/328 | 80.1% | 1.0% | 88.7% | 1.5% | 0.004 |
| ***„pain/discomfort“*** | | | | | | |
| **Total** | **1117** | **no: 45.7%. some: 49.9%. extreme: 4.5%** | | | | **-** |
| Female/male (total) | 528/589 | 38.3% | 5.5% | 52.3% | 3.6% | <0.001 |
| Female/male (intervention group) | 242/261 | 38.4% | 5.4% | 55.9% | 3.8% | <0.001 |
| Female/male (control group) | 286/328 | 38.1% | 5.6% | 49.4% | 3.4% | 0.014 |
| ***„anxiety/depression“*** | | | | | | |
| **Total** | **1120** | **no: 71.2%. some: 27.0%. extreme: 1.9%** | | | | **-** |
| Female/male (total) | 532/588 | 63.2% | 2.8% | 78.4% | 1.0% | <0.001 |
| Female/male (intervention group) | 242/262 | 63.2% | 3.3% | 81.3% | 0.4% | <0.001 |
| Female/male (control group) | 290/326 | 63.1% | 2.4% | 76.1% | 1.5% | 0.002 |

^1^ Percentage adding up to 100 percent means “some problems”

^2^ Fisher’s exact test or chi-square-test, respectively
