# Supplementary material for: The impact of a disease management programme for type 2 diabetes on health-related quality of life: multilevel analysis of a cluster-randomised controlled trial
Source: Diabetol Metab Syndr. 2018 Apr 10;10:28. doi: 10.1186/s13098-018-0330-9 (PMC5892002; doi:10.1186/s13098-018-0330-9)
Supplement: Supplementary file 6 — Additional file 6. EQ-index in the intervention group at follow-up, various subgroups disaggregated by sex. [file 13098_2018_330_MOESM6_ESM.docx]

Additional file 6 EQ-index in the intervention group at follow-up, various subgroups disaggregated by sex

|  | **Number of participants** | **EQ-index, mean difference ± SD** | | **p-value** | | |
| --- | --- | --- | --- | --- | --- | --- |
| Total | 470 | 0.00 ± 0.18 | | 0.612 | | |
| **Subgruppen** | **Female/**  **male** | **Female, mean ± SD^1^** | **Male, mean ± SD^1^** | **p-value^1^** | **p-value^2^** | **p-value^3^** |
| Female/male | 227/243 | -0.01 ± 0.19 | 0.01 ± 0.17 | 0.641 | 0.244 | <0.001 |
| Non Austrian | 10/15 | -0.07 ± 0.28 | -0.03 ± 0.18 | 0.303 | 0.665 | 0.447 |
| Austrian | 217/228 | -0.00 ± 0.19 | 0.02 ± 0.17 | 0.425 | 0.253 | <0.001 |
| Living with a partner | 147/207 | -0.00 ± 0.17 | 0.01 ± 0.16 | 0.898 | 0.528 | <0.001 |
| Living alone | 77/31 | -0.00 ± 0.22 | 0.04 ± 0.21 | 0.477 | 0.371 | 0.836 |
| High education (school leaving examination or higher) | 210/210 | -0.01 ± 0.20 | 0.01 ± 0.17 | 0.918 | 0.392 | <0.001 |
| No higher education | 14/31 | 0.01 ± 0.10 | 0.05 ± 0.15 | 0.214 | 0.374 | 0.750 |
| Not working fulltime | 206/195 | -0.00 ± 0.19 | 0.01 ± 0.17 | 0.540 | 0.446 | <0.001 |
| Working fulltime | 20/48 | -0.05 ± 0.18 | 0.02 ± 0.17 | 0.542 | 0.168 | 0.227 |
| Non-smoker | 201/204 | -0.01 ± 0.19 | 0.02 ± 0.16 | 0.556 | 0.122 | <0.001 |
| Current smoker | 26/39 | 0.01 ± 0.20 | -0.01 ± 0.19 | 0.991 | 0.554 | 0.039 |
| No manifestation of coronary heart disease^4^ | 199/200 | -0.00 ± 0.19 | 0.01 ± 0.16 | 0.819 | 0.674 | <0.001 |
| Any manifestation of coronary heart disease | 28/43 | -0.04 ± 0.23 | 0.05 ± 0.21 | 0.772 | 0.104 | 0.292 |
| No macrovascular diabetic complication^5^ | 174/177 | -0.00 ± 0.17 | 0.01 ± 0.15 | 0.741 | 0.587 | <0.001 |
| Any macrovascular diabetic complication | 53/66 | -0.02 ± 0.25 | 0.03 ± 0.21 | 0.796 | 0.251 | 0.023 |
| Non guideline adherence treatment^6^ | 120/123 | -0.01 ± 0.21 | 0.01 ± 0.19 | 0.913 | 0.556 | 0.006 |
| Full guideline adherence treatment^6^ | 107/120 | -0.01 ± 0.17 | 0.02 ± 0.14 | 0.530 | 0.250 | 0.002 |

^1^ Within-subject factor (time)

^2^ Within-subject factor (time*sex)

^3^ Between subject factors (sex)

^4^ Myocardial infarction and/or PTCA/stenting and/or coronary bypass

^5^ Myocardial infarction and/or PTCA/stenting and/or coronary bypass and/or stroke and/or carotid surgery and/or amputation/gangrene and/or peripheral artery bypass or PTA

^6^ A full guideline adherence treatment covered four aspects: patient education; diagnostic measures (i.e. regular HbA1c-checks); ophthalmological; and foot examinations
